# Hospital Meetings

**Published:** 1905-12-09

**Authors:** 


### HOSPITAL MEETINGS.

### MIDDLESEX HOSPITAL.

The quarterly Court of Governors was held at the hospital on Thursday, November 30.

There was a large attendance and the chair was taken by Lord Sandhurst.

Lord Cheylesmore, Chairman of the Weekly Board, in moving the adoption of the quarterly report, remarked that the hospital had been closed for two months in order to carry out some repairs which were quite imperative, the roof, for example, not being rainproof. The sum of £4,000 had been spent on these repairs. On the advice of the medical officers they had also provided a complete sterilising plant in connection with the operating theatres at a cost of £1,000. The Board much regretted having to receive the resignation of the Senior Surgeon, Mr. Henry Morris, who had so generously given £1,000 towards the establishment of a Permanent Endowment Fund for the maintenance of the medical school. The court would be asked to elect Mr. Morris a consulting surgeon to the hospital. They had also accepted, with the utmost regret, the resignation of Miss Thorold, who had held the post of Lady Superintendent for thirty-five years and had always performed her duties with the utmost zeal and devotion. The court would be asked to confirm the recommendation of the Board to grant Miss Thorold a pension of £200 a year in recognition of the admirable and conscientious work which she had accomplished during her long tenure of office, which was evidenced by the high standard of efficiency to which she had raised the nursing staff. Miss Eleanor C. Vernet had been appointed to fill Miss Thorold's place. The number of patients treated during the four months the hospital was open had been 1,297. The cancer wards had also been closed for two months for repairs. Dr. Lazarus Barlow had been reappointed Director of the Cancer Research Laboratories. The sum of £333 had been received from the Hospital Sunday Fund for the Cancer Charity. They had been authorised by the governors to set aside £2,000 for the maintenance of the Medical School, and Mr. H. Morris's generosity had enabled them to open a separate Endowment Fund, towards which contributions were earnestly invited.

The adoption of the report was seconded by Mr. Arthur Watson.

Lord Sandhurst then said a few words in recognition of the admirable manner in which the honorary staff performed their duties.

A vote of thanks having been passed to the Chairman, the whole party adjourned to witness the handsome presentation of plate and jewellery which was made to Miss Thorold by the Governors, members of the honorary medical and surgical staff, past and present sisters and nurses and students of the hospital.

